# 

**Published:** 1849-01-01

**Authors:** 


### CONTENTS OF VOL. II.

### Analytical Reviews.

| | PAGE |
|---|---|
| Lallemand on the Psychology of the Reproductive Functions | 1 |
| On Deformity of the Infantile Cranium | 30 |
| Dr. Duke on the Cerebral Affections of Infancy and Childhood | 41 |
| German Pantheism: The Theory of Life | 58 |
| The Phenomena of Death | 76 |
| Sir George Lefevre on the Pathology of the Nerves and Nervous Maladies | 90 |
| Bloomingdale Asylum, New York | 189 |
| Calmeil on Mental Epidemics | 221 |
| Hamilton Labatt on the Use and Abuse of Restraint in the Treatment of the Insane | 240 |
| The Insanity of Men of Genius | 262 |
| Smee on Electro-Biology | 292 |
| The Nervous System | 302 |
| On the Insanity of Dean Swift | 349 |
| On Sleep and Sleeplessness | 373 |
| The State of Lunacy in Ireland | 388 |
| Lunatic Asylums and Insanity in America | 398 |
| On the Management of Hanwell Lunatic Asylum | 418 |
| The Unpublished MSS. of the late Alfred Wigan, M.D. | 497 |
| Dr. Mayo on Popular Superstitions | 512 |
| On the Pathology and Characteristics of Insanity | 534 |
| Dr. Holland on the Philosophy of Animated Nature | 555 |
| The Lord Chief Baron and the Nottidge Case | 564 |

### Original Communications.

| | |
|---|---|
| Dr. Winslow on the Effect of Solitary Confinement on the Mind | 115 |
| Dr. Michéa on the Blood in the Neuroses | 118 |
| Works and Memoirs on General Psychology | 134 |
| The Connexion between Physiology, Psychology, Natural Theology, and other Sciences | 309 |
| The Sedative Treatment of Insanity | 319 |
| Lord Brougham on Partial Insanity | 323 |
| Transient Insanity | 329 |
| Juvenile Delinquency and Degeneration in the Upper Classes of Society | 428 |
| Case of Double Consciousness consciousness connected with Hysteria | 456 |
| Supposed Demoniacal Possession | 462 |
| Impulsive Insanity.—The French Vampire | 577 |
| Madness, as treated by Shakspere | 589 |

### Selections.

| | |
|---|---|
| The Pulse of the Insane | 143 |
| Compression of the Carotids in Insanity | 144 |
| Treatment of Epilepsy | 144 |
| Lunatic Asylum, Nantes | 147 |
| Attempt at Murder: Insanity | 152 |
| Infanticide | 152 |
| Cerebral and Meningeal Phlebitis in Puerperal Women | 153 |
| Treatment of Epilepsy | 154 |
| Nocturnal Neuralgia of the Forearm | 155 |
| Paralysis independent of Lesion of the Nervous Centres | 155 |
| Œsophageal Catheterism of the Insane | 156 |
| Medico-Legal Report of the State of Mind of Marie Madeleine Droin | 157 |
| Decrease of Brain in Insanity | 159 |
| On Cerebral Congestion, considered in Relation to Hemorrhage and Softening of the Brain | 159 |
| Epileptiform Hysteria | 160 |
| Palsy of the Tongue cured by Galvano-Puncture | 162 |
| Apparent Death | 162 |

PAGE
Insanity ... 163
Acute Mania ... 163
Epilepsy ... 165
Cold Affusion ... 165
Treatment of Delirium Tremens ... 165
Gastralgia and Hypochondriasis... 165
The Organization of Labour for the Insane ... 167
Acute Tubercular Meningitis in the Adult ... 168
Complete Paraplegia cured by Cold Bathing and Urtication... 168
Chloroform in Tetanus ... 168
Melancholy cured by severe Bodily Injuries ... 169
Musk and Blisters in Acute Hydrocephalus ... 169
Mercurial Frictions in Encephalitis 170
Intermittent Cephalea caused by an Effusion of Blood between the Dura Mater and its Parietal Arachnoid ... 170
Aldehide ... 170
Action of Cannabis Indica ... 171
Periodic Epilepsy, Hysteria, Neuralgia, and Hemiplegia ... 171
Chorea in Scrofulous Subjects ... 171
Influence of the Penitentiary System on Insanity ... 171
Eclampsia cured by Stramonium... 172
Tetanus cured by Intoxication ... 173
Effect of Music on the Nerves ... 173
Medico-Legal Investigation ... 344
Danger of too frequent Abstraction of Blood in the General Palsy of the Insane ... 346
Suicides in France ... 347
Chloroform in Fractional Doses... 347

### Medical Jurisprudence.

The Plea of Insanity... 331
Commissions in Lunacy... 468

### Correspondence.

Letters from Paris ... 338, 624

### Medical Intelligence and News.

The Study of Mental Diseases ... 174
Puerperal Insanity ... 175
Law of Lunacy...178, 186
Literary Notices ... 188
Books received... 188, 348
PAGE
Death of Dr. Prichard... 188
Appointments ... 348
Judicial Insanity—Trial of Nottidge *v.* Ripley ... 630

### Psychological Fragments.

Progressive General Palsy ... 613

Hallucinations in Early Infancy ... 616

Prolonged Bathing and Continuous Irrigation in the Treatment of Acute Forms of Insanity ... 616

Endemic Cerebro-Spinal Meningitis 617

Experiments on the Respiratory and Arterial Motions of the Brain ... 617

Hysteria ... 617

Treatment of Hysteria and Chlorosis 618

Chronic Sciatica ... 618

Sonnambulism... 618

Aconitum Napellus ... 618

Chloroform in Neuralgia... 619

Treatment of Epilepsy ... 619

Hydrophobia ... 619

Epilepsy ... 619

The Essential Pathological Conditions of the Brain in Insanity ... 620

Hemicrania ... 622

Origin of the Vis Nervosa ... 622

Chloroform in Delirium Tremens ... 622

Chloroform in Quotidian Hemicrania 622

Puerperal Convulsions ... 622

Lunatic Asyla in the United States 623

### Miscellaneous.

Remarks on the Causes and Morbid Anatomy of Mental Diseases ... 483
The New County Asylum for Middlesex, Colney Hatch ... 490
The Study of Mental Diseases ... 495
Notice to Correspondents ... 496
Letter to the Lord Chancellor from the Commissioners in Lunacy ... 608
Books and Journals received for Review ... 635
Journals in Exchange... 636

---

MONOGRAPH II.—On Softening of the Brain, arising from Anxiety and Undue Mental Exercise, and resulting in Impairment of Mind. By FORBES WINSLOW, M.D.

